# LOCC indistinguishable orthogonal product quantum states

**DOI:** 10.1038/srep28864

**Published:** 2016-07-05

**Authors:** Xiaoqian Zhang, Xiaoqing Tan, Jian Weng, Yongjun Li

**Affiliations:** 1 Department of Mathematics, Jinan University, Guangzhou, P.R. China; 2 Department of Computer Science, Jinan University, Guangzhou, P.R. China; 3 School of Computer Science and Engineering, South China University of Technology, Guangzhou, P.R. China

## Abstract

We construct two families of orthogonal product quantum states that cannot be exactly distinguished by local operation and classical communication (LOCC) in the quantum system of 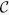
^2*k*+*i*^ ⊗ 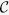
^2*l*+*j*^ (*i, j* ∈ {0, 1} and *i* ≥ *j* ) and 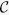
^3*k*+*i*^ ⊗ 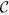
^3*l*+*j*^ (*i, j* ∈ {0, 1, 2}). And we also give the tiling structure of these two families of quantum product states where the quantum states are unextendible in the first family but are extendible in the second family. Our construction in the quantum system of 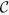
^3*k*+*i*^ ⊗ 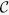
^3*l*+*j*^ is more generalized than the other construction such as Wang *et al*.’s construction and Zhang *et al*.’s construction, because it contains the quantum system of not only 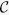
^2*k*^ ⊗ 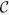
^2*l*^ and 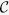
^2*k*+1^ ⊗ 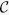
^2*l*^ but also 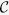
^2*k*^ ⊗ 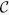
^2*l*+1^ and 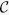
^2*k*+1^ ⊗ 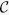
^2*l*+1^. We calculate the non-commutativity to quantify the quantumness of a quantum ensemble for judging the local indistinguishability. We give a general method to judge the indistinguishability of orthogonal product states for our two constructions in this paper. We also extend the dimension of the quantum system of 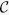
^2*k*^ ⊗ 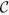
^2*l*^ in Wang *et al*.’s paper. Our work is a necessary complement to understand the phenomenon of quantum nonlocality without entanglement.

In quantum cryptography, quantum entangled states are easily distinguished by performing global operation if and only if they are orthogonal. Entanglement has good effects in some cases, but it has bad effects in other cases such as entanglement increases the difficulty of distinguishing quantum states when only LOCC is performed^1^. When many global operations cannot be performed, LOCC becomes very useful. The phenomenon of quantum nonlocality without entanglement^2^ is that a set of orthogonal states in a composite quantum system cannot be reliably distinguished by LOCC. The study of quantum nonlocality is one of the fundamental problems in quantum information theory. LOCC is usually used to verify whether quantum states are perfectly distinguished^3^,^4^,^5^,^6^,^7^,^8^,^9^,^10^,^11^,^12^,^13^,^14^,^15^,^16^,^17^,^18^,^19^,^20^,^21^,^22^,^23^ or not. In refs 3, 4, 5, 6, 7, 8, 9, 10, 11, 12, they focus on the local distinguishability of quantum states such as multipartite orthogonal product states can be exactly distinguished^10^ or how to distinguish two quantum pure states^11^,^12^. Moreover, locally indistinguishability^13^,^14^,^15^,^16^,^17^,^18^,^19^,^20^,^21^,^22^,^23^ of quantum orthogonal product states plays an important role in exploring quantum nonlocality.

The nonlocality problem is considered in the bipartite setting case that Alice and Bob share a quantum system which is prepared in an known set contained some mutually orthogonal quantum states. Their aim is to distinguish the states only by LOCC. Bennett *et al*.^2^ proposed a set of nine pure orthogonal product states in quantum system of *C*^3^ ⊗ *C*^3^ in 1999, which cannot be exactly distinguished by LOCC. In 2002, Walgate *et al*.^16^ also proved the indistinguishability of the nine states by using a more simple method. Zhang *et al*.^19^ extended the dimension of quantum system in Walgate *et al*.’s^16^. Yu and Oh^22^ give another equivalent method to prove the indistinguishability and this method is used to distinguish orthogonal quantum product states of Zhang *et al*.^21^. Furthermore, Wang *et al*.^20^ constructed orthogonal product quantum states under three quantum system cases of 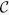
^2*k*^ ⊗ 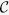
^2*l*^, 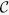
^2*k*^ ⊗ 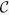
^2*l*+1^ and 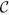
^2*k*+1^ ⊗ 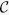
^2*l*+1^. The smallest dimension of 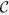
^2*k*^ ⊗ 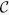
^2*l*^ can be constructed is 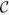
^6^ ⊗ 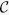
^6^ in Wang *et al*.’s paper^20^. However, the smallest dimension of 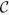
^2*k*^ ⊗ 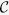
^2*l*^ can be constructed is 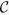
^4^ ⊗ 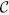
^4^ in our paper. Ma *et al*.^24^ revealed and established the relationship between the non-commutativity and the indistinguishability. By calculating the non-commutativity, the quantumness of a quantum ensemble can be quantified for judging the indistinguishability of a family of orthogonal product basis quantum states. For the orthogonal product states, we firstly use a method to judge the indistinguishability of the set, the proof is meaningful. In this paper, we calculate the non-commutativity to judge the indistinguishability if and only if there exists a set to satisfy the inequality of *Lemma 2*.

In this paper, we construct two families of orthogonal product quantum states in quantum systems of 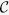
^2*k*+*i*^ ⊗ 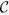
^2*l+j*^ with *i, j* ∈ {0, 1} (*i* ≥ *j*) and 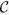
^3*k*+*i*^ ⊗ 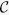
^3*l*+*j*^ with *i, j* ∈ {0, 1, 2} and the two families of orthogonal product quantum states cannot be exactly distinguished by LOCC but can be distinguished by separable operations. Our constructions give the smaller dimension of quantum system in quantum system of 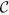
^2*k*^ ⊗ 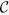
^2*l*^ than Wang *et al*.’s^20^. Wang *et al*.’s construction can be extended, but our construction in quantum system of 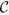
^2*k*+*i*^ ⊗ 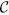
^2*l*+*j*^ with *i, j* ∈ {0, 1} (*i* ≥ *j*) is a complete unextendible product bases (i.e. UPB). Therefore, our construction is trivial. The indistinguishability of a complete UPB can be directly judged by performing projective measurements and classical communication, but not Wang *et al*.’s^20^. In quantum system of 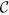
^3*k*+*i*^ ⊗ 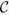
^3*l*+*j*^ (*i, j* = 0, 1, 2), it contains not only 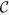
^2*k*^ ⊗ 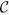
^2*l*^ and 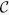
^2*k*+1^ ⊗ 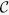
^2*l*^ but also 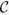
^2*k*^ ⊗ 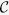
^2*l*+1^ and 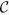
^2*k*+1^ ⊗ 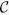
^2*l*+1^, so our construction in quantum system of 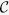
^3*k*+*i*^ ⊗ 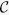
^3*l*+*j*^ with *i, j* ∈ {0, 1, 2} is more generalized than Zhang *et al*.^19^ and Wang *et al*.^20^. We also use a simple method to judge the local indistinguishable by calculating the non-commutativity to quantify the quantumness of a quantum ensemble^24^, but not Zhang *et al*. and Wang *et al*. We also generalize the Theorem 2 in Ma *et al*.^24^ to *Corollary 1* in Methods in this paper. Our work is a necessary complement to understand the phenomenon of quantum nonlocality without entanglement.

## Results

### LOCC indistinguishable orthogonal product quantum states in quantum system of 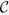 ^2*k*+*i*^ ⊗ 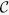 ^2*l*+*j*^ with *k* ≥ 1, *l *≥ 1 and *i, j* ∈ {0, 1} (*i* ≥ *j*)

**Figure 1:**
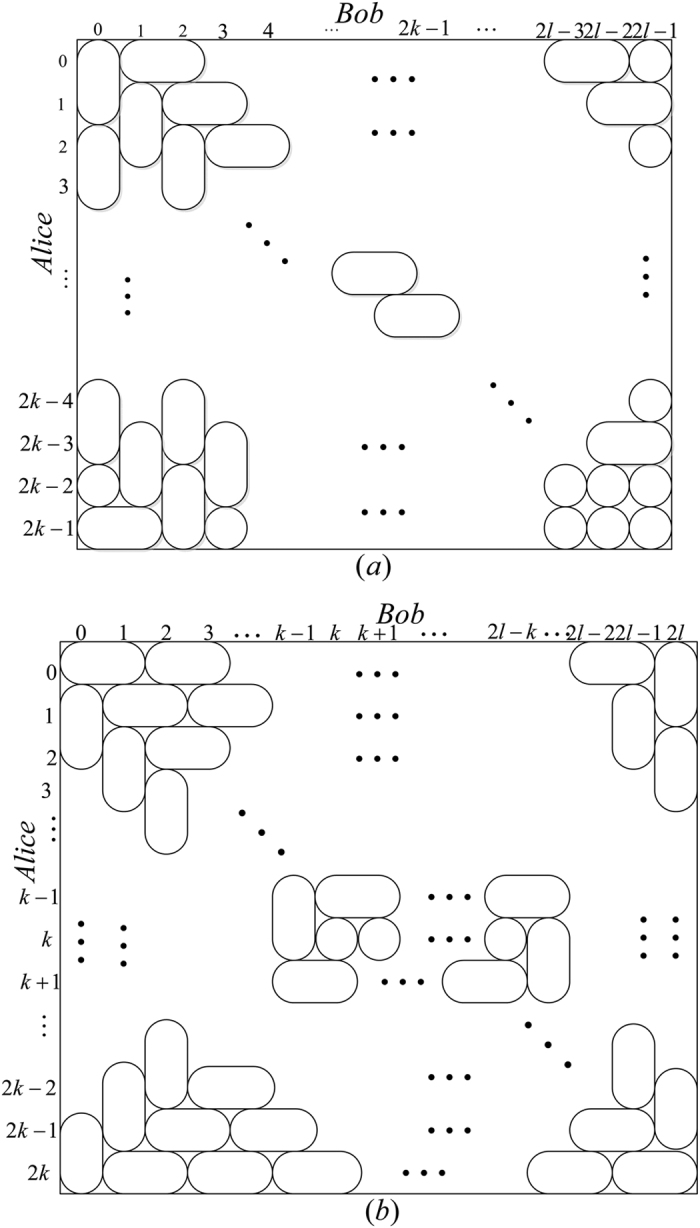
The tiling structure of orthogonal product quantum states in quantum system of (**a**) 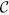
^2*k*^ ⊗ 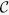
^2*l*^ with *k, l* ≥ 2 and (**b**) 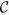
^2*k*+1^ ⊗ 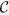
^2*l*+1^ with *k, l* ≥ 1.

**Figure 2:**
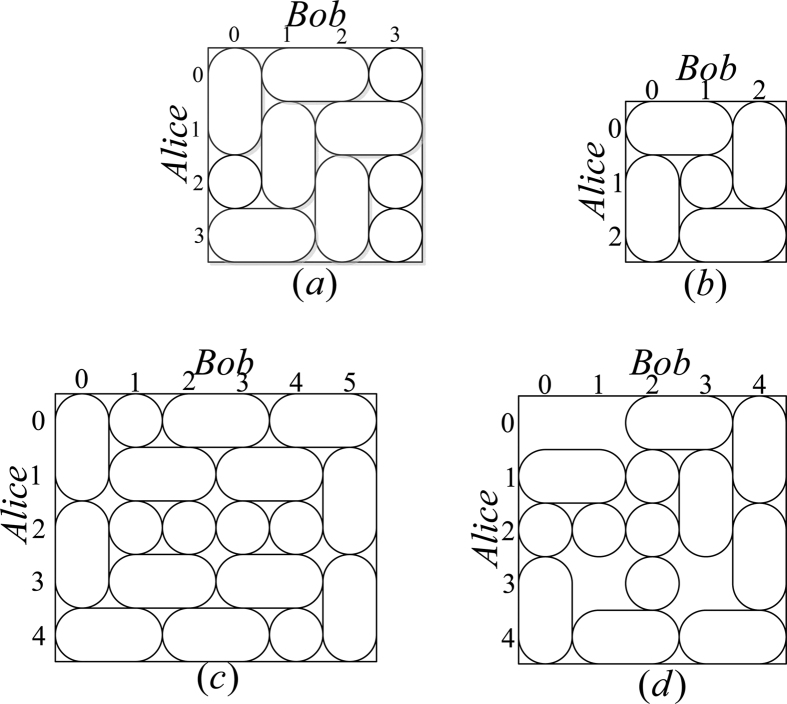
The tiling structure of orthogonal product quantum states in quantum system of (**a**) 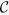
^4^ ⊗ 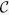
^4^, (**b**) 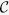
^3^ ⊗ 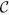
^3^, (**c**) 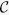
^5^ ⊗ 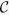
^6^, and (**d**) 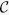
^5^ ⊗ 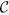
^5^.

*Case 1*. Firstly, we construct LOCC indistinguishable orthogonal product quantum states in quantum system of 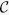
^2*k*^ ⊗ 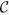
^2*l*^ (*k, l* ≥ 2) (see Fig. 1(a)) and give an example in the smallest dimension (see Fig. 2(a)).


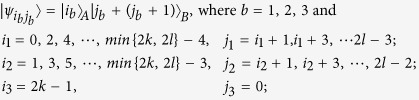



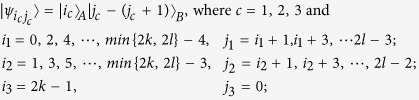



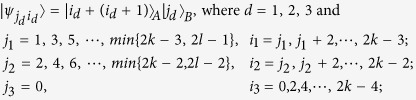



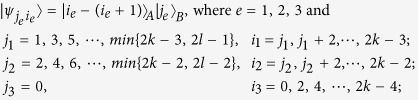



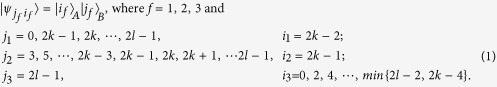


Here 
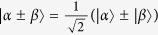
. For example, 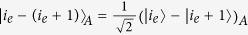.

*Proposition 1. In quantum system of* 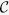
^2*k*^ ⊗ 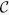
^2*l*^, *there are* 4 *kl orthogonal product quantum states* |*ψ*_*i*_〉 (*in* Eq. (1)) *can not be exactly distinguished by LOCC whatever Alice measures firstly or Bob*.

*Proof*. We only discuss the case of Alice measures firstly and the same as Bob. We consider the subspace 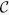
^2^ ⊗ 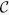
^*m*^ to determine POVM elements 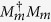. A set of general 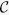
^2*k*^ ⊗ 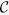
^2*k*^ POVM elements 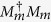 under the basis 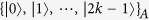 can be expressed as follows


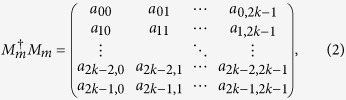


where *a*_*ij*_ ≥ 0 and 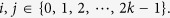 Firstly, this selected sets {|0〉, |1〉}_*A*_, {|1〉, |2〉}_*A*_, …, and {|2*k* − 2〉, |2*k* − 1〉}_*A*_ of states are of dimension 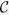
^2^ ⊗ 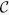
^2*k*^, Alice cannot find appropriate basis to express them in the form of Eq. (23) in Methods according to the necessary and sufficient condition of *Lemma 1*.

For example, we consider the subspace {|0〉, |1〉}_*A*_, there are quantum states


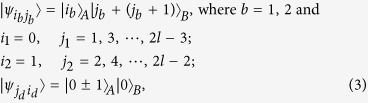


The necessary and sufficiency of *Lemma 1* has already been proved by Walgate in ref. 16. Now we apply the necessary and sufficiency of *Lemma 1* to verify *a*_00_ = *a*_11_ and *a*_10_ = *a*_01_ = 0 in the subspace {|0〉, |1〉}_*A*_. Suppose, the form 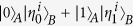 is set up in Eq. (23), where 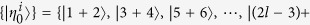

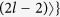
, 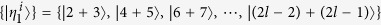. The two sets 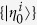 and 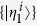 satisfy 
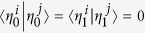
 if *i* ≠ *j*. However, there also exist quantum states |0 ± 1〉_*A*_ in the subspace {|0〉, |1〉}_*A*_. The reduction to absurdity is used to verify the correctness of the conclusion. Suppose there exists one POVM element that is not proportional to identity to distinguish these quantum states, the express of the POVM element is as follows


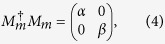


where *α* > *β* ≥ 0. For the quantum state |0〉_*A*_, it collapses into *α*|0〉_*A*_ after measurement. For the quantum state |1〉_Α_, it collapses into *β*|1〉_*A*_ after measurement. For the quantum states 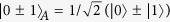, they collapse into 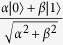. Hence, if and only if *α* = *β*, 
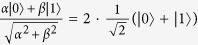
 holds. It produces contradiction between results and assumption. So it does not exist a non-trivial measurement to distinguish the orthogonal product quantum states. For the other subspaces, we have the same conclusions. After Alice performs a general measurement, the effect of this positive operator upon the following states


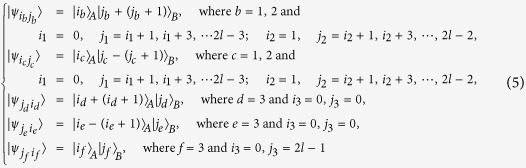


is entirely specified by those elements *a*_00_, *a*_11,_ *a*_01,_ *a*_10_ draw from the {|0〉, |1〉}_*A*_ subspace. It means that Alice cannot perform a nontrivial measurement upon the {|0〉, |1〉}_*A*_ subspace. Thus, the corresponding submatrix must be proportional to the identity. Then, we obtain *a*_00_ = *a*_11_ = *a, a*_01_ = *a*_10_ = 0. For the states


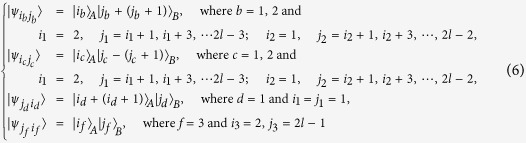


and the subspace {|1〉, |2〉}_*A*_, we make the same argument. Then we get the result *a*_11_ = *a*_22_ = *a, a*_12_ = *a*_21_ = 0. For the states


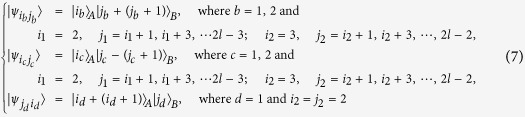


and subspace {|2〉, |3〉}_*A*_, we get the result *a*_22_ = *a*_33_ = *a, a*_23_ = *a*_32_ = 0. In the same way, for the subspace {|3〉, |4〉}_*A*_, …, the subspace 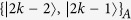, we get the result


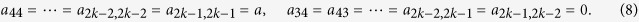


Because POVM elements 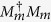 is Hermitian, the equation 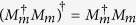 is correct. Then we obtain


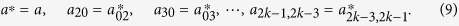


Now 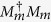 can be rewritten as


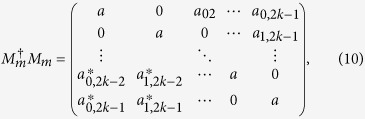


where *a* is a real number.

We now consider the states 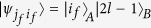 with *f* = 3, *i*_3_ = 0, 2 and the subspace {|0〉, |2〉}_*A*_. After Alice measures, the result is either the states orthogonal or distinguishing them outright. If the states are orthogonal, we demand that 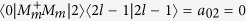. So, we get 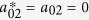. For the states 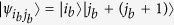 with *i*_*b*_ = 3, *j*_*b*_ = 2*l*−2 and 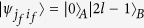, we get the same argument and we get 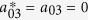. For the subspace {|0〉, |4〉}_*A*_, {|0〉, |5〉}_*A*_, … and the subspace {|2*k* − 3〉, |2*k* − 1〉}_*A*_, we get the results


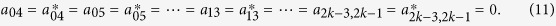


Now the 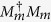 is proportional to the identity. However, if Alice distinguishes the state 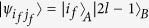 with *f* = 3, *i*_*f*_ = 0, 2, we get the result 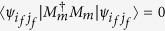. We can also have the result 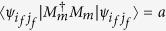, therefore *a* = 0. It produces contradictory with the theorem of Walgate^16^. So, 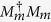 is proportional to the identity and the 4*kl* orthogonal product states are indistinguishable.

*Example 1*. Now we will give 16 orthogonal product quantum states in quantum system of 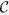
^4^ ⊗ 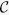
^4^ (see Fig. 2(a)).


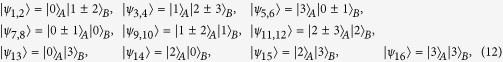


where 
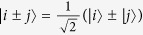
 with 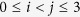.

*Case 2*. Secondly, we construct LOCC indistinguishable orthogonal product quantum states in quantum system of 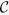
^2*k*+1^ ⊗ 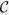
^2*l*+1^ with *k, l* ≥ 1 and *l* ≤ *l* (see Fig. 1(b)) and also give an example in the smallest dimension (see Fig. 2(b)).


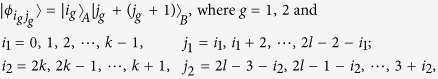



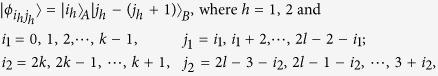



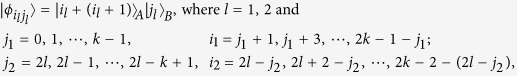



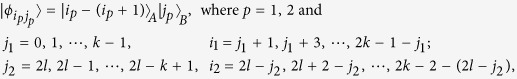



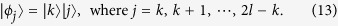


Here we just give the construction for *k* ≤ *l*. When *k* > *l*, it should be rotated along the clockwise direction for Fig. 1(b) to get the construction.

*Proposition 2. In quantum system of* 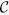
^2*k*+1^ ⊗ 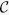
^2*l*+1^, *there are* (2*k* + 1)(2*l* + 1) *orthogonal product quantum states* |*ϕ*_*i*_〉 (*in* Eq. (13)) *can not be exactly distinguished by LOCC whatever Alice measures firstly or Bob*.

*Example 2*. Now we will give 9 orthogonal product quantum states in quantum system of 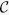
^3^ ⊗ 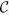
^3^ (see Fig. 2(b)).


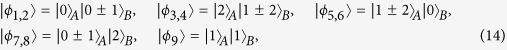


where 
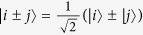
 with 0 ≤ *i* < *j* ≤ 2.

**Figure 3:**
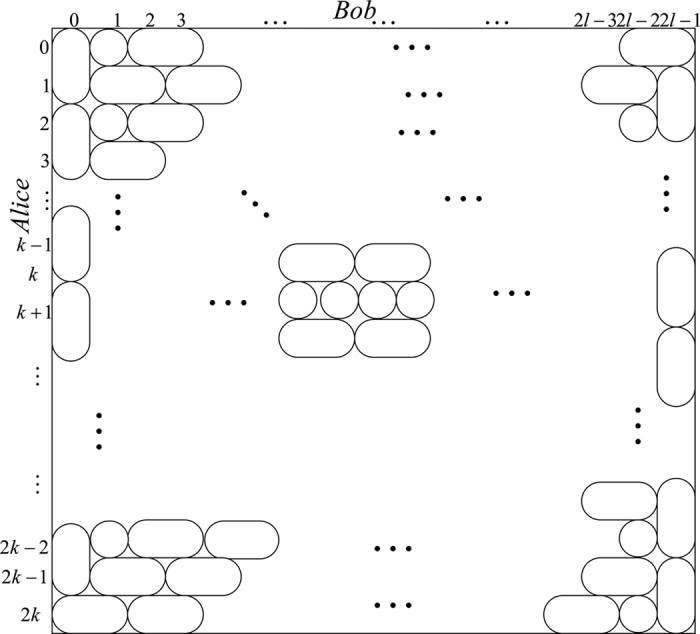
The tiling structure of orthogonal product quantum states in quantum system of 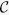
^2*k*+1^ ⊗ 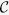
^2*l*^ with *k* ≥ 2, *l* ≥ 3.

*Case 3*. Thirdly, we consider the indistinguishable orthogonal product states in quantum system 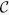
^2*k*+1^ ⊗ 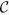
^2*l*^ with *k* ≥ 2, *l* ≥ 3 (see Fig. 3) and give an example in the smallest dimension (see Fig. 2(c)).


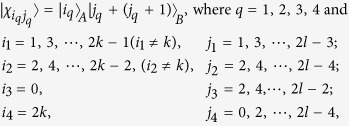



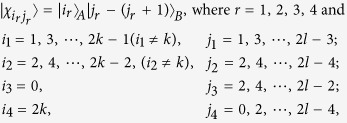



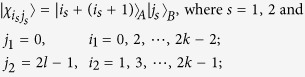



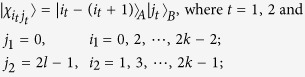



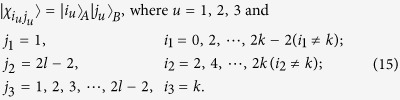


*Proposition 3. In quantum system of* 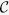
^2*k*+1^ ⊗ 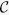
^2*l*^, *there are* 2*l*(2*k* + 1) *orthogonal product quantum states* 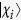 (*in* Eq. (15)) *can not be exactly distinguished by LOCC whatever Alice measures firstly or Bob*.

*Example 3*. Now we will give 30 orthogonal product quantum states in quantum system of 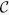
^5^ ⊗ 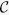
^6^ (see Fig. 3(c)).


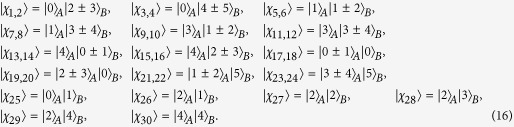


where 
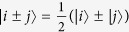
 with 0 ≤ *i* ≤ 4 and 0 ≤ *j* ≤ 5.

### LOCC indistinguishable orthogonal product quantum states in quantum system of 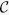 ^3*k*+*i*^ ⊗ 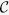 ^3*k*+*j*^ with *i, j* ∈ {0, 1, 2}

We give LOCC indistinguishable orthogonal product quantum states in quantum system of 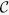
^*m*^ ⊗ 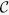
^*n*^.


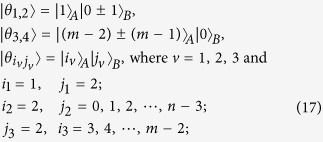


In quantum system of 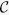
^3*k*^ ⊗ 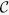
^3*l*^, 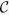
^3*k*^ ⊗ 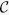
^3*l*+*2*^, 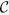
^3*k*+*2*^ ⊗ 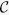
^3*l*^ with *k, l* ≥ 2, 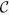
^3*k*+*2*^ ⊗ 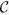
^3*l*+*2*^ with *k, l* ≥ 1, 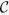
^3*k*+1^ ⊗ 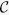
^3*l*^ and 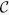
^3*k*+1^ ⊗ 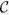
^3*l*+2^ with *k, l* ≥ 2.


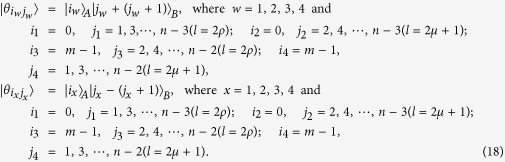


In quantum system of 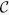
^*m*^ ⊗ 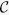
^*n*^ including 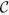
^3*k*^ ⊗ 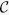
^3*l*+1^, 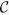
^3*k*+2^ ⊗ 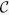
^3*l*+1^, 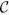
^3*k*+1^ ⊗ 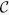
^3*l*+1^ with *k, l* ≥ 2.


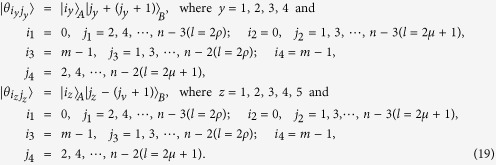


In quantum system of 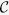
^*m*^ ⊗ 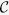
^*n*^ including 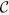
^3*k*^ ⊗ 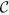
^3*l*^, 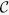
^3*k*^ ⊗ 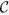
^3*l*+2^, 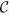
^3*k*+2^ ⊗ 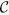
^3*l*^, 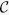
^3*k*^ ⊗ 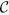
^3*l*+1^ and 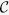
^3*k*+2^ ⊗ 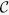
^3*l*+1^ with 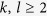, 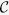
^3*k*+2^ ⊗ 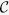
^3*l*+2^ with 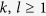.


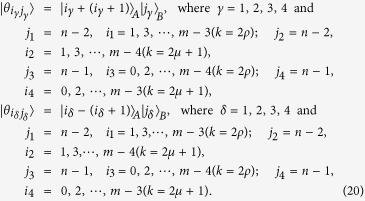


In quantum system of 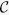
^*m*^ ⊗ 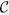
^*n*^ including 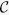
^3*k*+1^ ⊗ 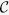
^3*l*^, 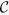
^3*k*+1^ ⊗ 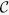
^3*l*+2^, 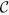
^3*k*+1^ ⊗ 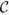
^3*l*+1^ with *k, l* ≥ 2.


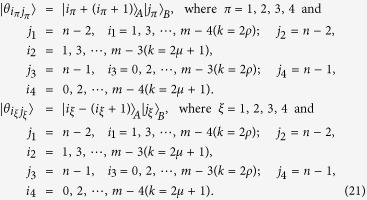


The equation *k* = 2*ρ* (or *k* = 2*μ* + 1) expresses that *k* is even (or odd).

*Proposition 4. In quantum system of* 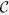
^*m*^ ⊗ 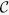
^*n*^, *there are* 3(*n* + *m*) − 9 *orthogonal product quantum states* |*θ*_*i*_〉 (*in* Eqs (17, 18, 19, 20, 21)) *can not be exactly distinguished by LOCC whatever Alice measures firstly or Bob, where m* = 3*k* + *i, n* = 3*l* + *j with i, j* ∈ {0, 1, 2}.

For the proof of the proposition 2, 3, 4, we make the same arguments to prove the indistinguishability only by LOCC. We only need to modify some relevant places.

*Example 4*. Now we will give the 21 orthogonal product quantum states in quantum system of 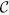
^5^ ⊗ 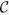
^5^ (see Fig. 2(d)).


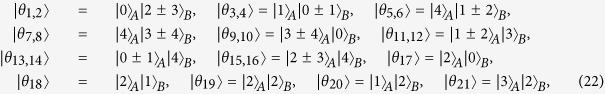


where 
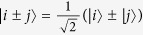
 with 0 ≤ *i* ≤ 4 and 0 ≤ *j* ≤ 4.

## Discussion

The orthogonal product quantum states constructed by us are indistinguishable by performing local operation and classical communication, but not separable operations^25^. Now, we discuss whether the separable operations can distinguish these product quantum states or not.

### LOCC indistinguishable orthogonal product quantum states in quantum system of 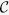 ^2*k*+*i*^ ⊗ 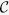 ^2*l*+*j*^ with *i, j* ∈ {0, 1} (*i* ≤ *j*)

Obviously, these states in Eqs (1, 12, 13, 14, 15, 16) can be distinguished by separable operations. The orthogonal quantum states are not extended. Suppose, the *mn* quantum states are 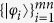 respectively. Now, we give the measurement operations 
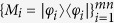
. Because the set 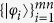 is an orthogonal product normal base of 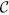
^*m*^ ⊗ 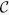
^*n*^, the equation 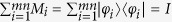 satisfies the completeness and 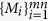 is a separable measurement. Due to 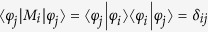, where 1 ≤ *i* ≤ *mn*, 1 ≤ *j* ≤ *mn*, if the measurement outcome is |*φ*_*i*_〉, the quantum state is |*φ*_*i*_〉. Therefore, the *mn* quantum states in Eqs (1, 12, 13, 14, 15, 16) can be distinguished by the separable operations.

Similar to Zhang *et al*.’s paper^19^, the multipartite quantum systems can be constructed when *m* = *n* = *d*. Such as in the quantum system 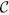
^*d*^ ⊗ 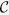
^*d*^ ⊗ 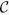
^*d*^, we give the orthogonal indistinguishable product states 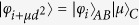, where 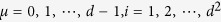 and 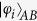 in Eqs (1, 12) of 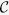
^2*k*^ ⊗ 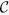
^2*l*^ and 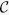
^2*k*+1^ ⊗ 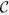
^2*l*+1^. However, Wang *et al*.’s construction^20^ cannot be extended into multipartite quantum systems because the set of orthogonal product states is extendible.

### LOCC indistinguishable orthogonal product quantum states in quantum system of 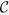 ^3*k*+*i*^ ⊗ 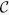 ^3*l*+*j*^ with *i, j* ∈ {0, 1, 2}

Similar to the first construction, the second construction is extendible and distinguished by separable operations. Firstly, these states in Eqs (17, 18, 19, 20, 21) all can be extended to *mn* orthogonal product states. Then, the proof of the process is the same as above. Finally, we construct the 3(*m* + *n*) − 9 quantum states respectively in Eqs (17, 18, 19, 20, 21) that can be distinguished by the separable operations.

## Methods

In ref. 16, Walgate *et al*. gave a necessary and sufficient condition to prove the local indistinguishability of a set of orthogonal product states. If a quantum system which is a qubit does not exist, a uniform conclusion cannot be drawn yet. In all LOCC protocols, there must be a party to leave.

*Lemma 1*^16^. *Alice and Bob share a* 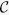
^2^ ⊗ 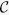
^*n*^ *dimensional quantum system: Alice has a qubit, and Bob has an n dimensional system that may be entangled with that qubit. If Alice goes first, a set of orthogonal states* {|*φ*_*i*_〉} *is exactly locally distinguishable if and only if there is a basis* {|0〉, |1〉}_*A*_ *such that in that basis*


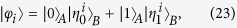


*where* 
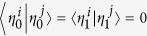
 if *i* ≠ *j*. The *Lemma 1* is used to prove the indistinguishability of 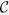
^2*k*+*i*^ ⊗ 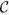
^2*l*+*j*^ quantum system with *i, j* ∈ {0, 1} (*i* ≤ *j*) and 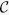
^3*k*+*i*^ ⊗ 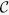
^3*l*+*j*^ quantum system with *i, j* ∈ {0, 1, 2} in Results.

*Definition*^24^. *Let* 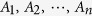 *be a set of operators. The total non-commutativity for this set is defined*


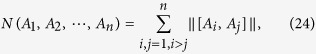


*where* [*A, B*] = *AB* − *BA*, 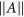 *is the trace norm of the operator A*, 
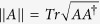
.

In the Methods of Ma *et al*.’s^24^, they give the concrete calculation formula, i.e. suppose 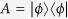 and 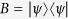. Denote 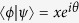 with *x* ∈ [0, 1], 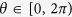. Hence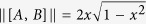. When 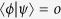 or 1, 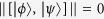, when 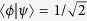, 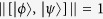 and when 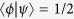, 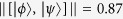. Nextly, we give *Lemma 2* as a standard of judging the indistinguishability of complete orthogonal product states.

*Lemma 2*^24^. *For a complete set of* 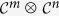 *POPS*, 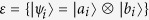 *with* 
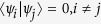
, *the ε cannot be completely locally distinguished if and only if there exist subsets* 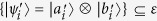, *such that* 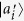 *and* 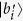 *are all single sets, i*.*e. there exist* 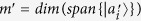 *linear independent* 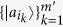 *in* 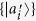 *and* 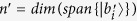 *linear independent* 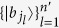 *in* 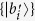 *satisfying*


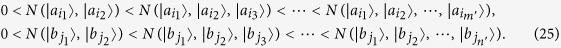


The quantity non-commutativity is used to quantify the quantumness of a quantum ensemble for judging the indistinguishability.

Here, we use the simply method in *Lemma 2* to judge the indistinguishability of orthogonal product states in^24^ by calculating the non-commutativity *N*. The orthogonal product quantum states in Eqs (1, 13, 15) are complete. Such as the set of complete orthogonal product states in Eq. (1), we give the briefly process. Firstly, we give the sets of *ε*^*A*^ and *ε*^*B*^.


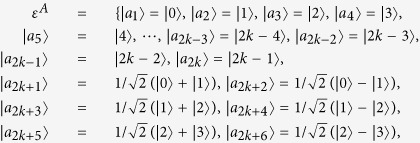



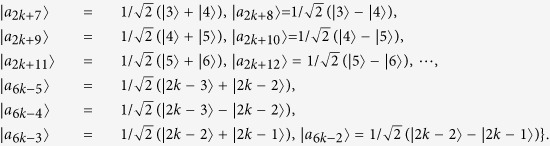



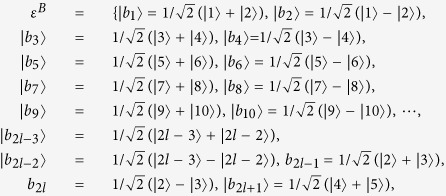



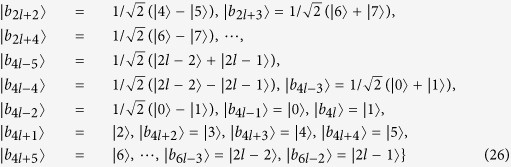


Some duplicate items are removed in *ε*^*A*^ and *ε*^*B*^. Nextly, we concretely calculate the non-commutativity *N* to quantify the quantumness of a quantum ensemble. There are 2*k* = (*spanε*^*A*^) linear independent states in *ε*^*A*^.


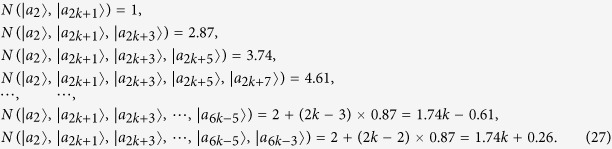


For the last two non-commutativity 1.74*k* + 0.26 and 1.74*k* + 0.61, we obtain that the difference (1.74*k* + 0.26) −(1.74*k* − 0.61) = 0.87 > 0. Hence, we obtain the inequality as follows


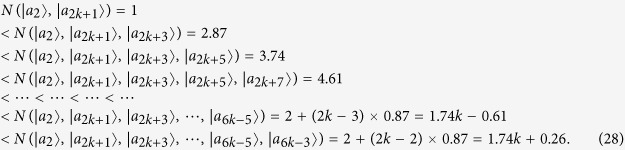


So *ε*^*A*^ is a single set according to Lemma 2.

There are 2*l* = *dim*(*spanε*^*B*^) linear independent states in *ε*^*B*^.


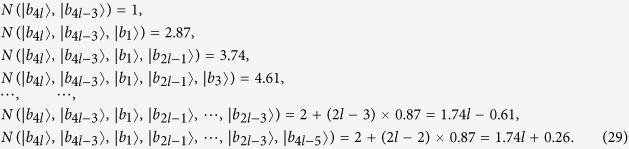


For the last two non-commutativity 1.74*l* + 0.26 and 1.74*l* + 0.61, we obtain that the difference (1.74*l* + 0.26)−(1.74*l* − 0.61) = 0.87 > 0. Hence, we obtain the inequality as follows


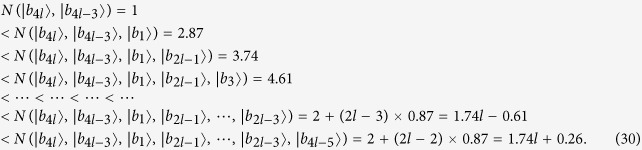


So *ε*^*B*^ is also a single set according to *Lemma 2*. According to the necessary and sufficient condition of *Lemma 2*, we make a conclusion that the set of complete orthogonal product quantum states in Eq. (1) is indistinguishable. Similarly, for the orthogonal product states in Eqs (13, 15), we obtain the same conclusion. The quantum orthogonal product states in Eqs (17, 18, 19, 20, 21) are incomplete but can be extended into a complete set, we can also judge the indistinguishability by *Corollary 1*. Now we will introduce the *Corollary 1*.

*Corollary 1*. For a incomplete set of orthogonal product states in quantum system of 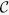
^*m*^ ⊗ 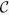
^*n*^, it firstly should be extended into a complete set 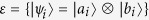 with 
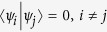
 if and only if it is completable. The indistinguishability of its complete set can be judged by *Lemma 2*.

The *Corollary 1* is used to judge the indistinguishability of a set of incomplete orthogonal product states which is completable. The second family construction in quantum system of 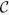
^3*k*+*i*^ ⊗ 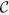
^3*l*+*j*^ with 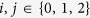 is incomplete but can be completable, so we can use the *Corollary 1* to judge the indistinguishability. For example, for the quantum system of 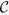
^3*k*^ ⊗ 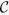
^3*l*^ when *k, l* are all even, quantum states |0〉_*A*_|0〉_*B*_, |1〉_*A*_|*n*_1_〉_*B*_ with *n*_1_ = 3, 4, 5, 6, …, 3*l* − 3, |*m*〉_*A*_|*n*_2_〉_*B*_ with 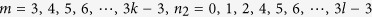, 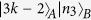 with 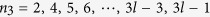 and |3*k* − 1〉_*A*_|2〉_*B*_ are added into the original incomplete set. The original incomplete set becomes a complete set. And its indistinguishability can be judged by using *Corollary 1*.

## Additional Information

**How to cite this article**: Zhang, X. *et al*. LOCC indistinguishable orthogonal product quantum states. *Sci. Rep.* **6**, 28864; doi: 10.1038/srep28864 (2016).
